# Practical Consequences of Item Response Theory Model Misfit in the Context of Test Equating with Mixed-Format Test Data

**DOI:** 10.3389/fpsyg.2017.00484

**Published:** 2017-04-04

**Authors:** Yue Zhao, Ronald K. Hambleton

**Affiliations:** ^1^Teaching and Learning Evaluation and Measurement Unit, The University of Hong KongHong Kong, Hong Kong; ^2^Department of Educational Policy, Research and Administration, University of Massachusetts, AmherstMA, USA

**Keywords:** item response theory (IRT), model misfit, model misspecification, test equating, IRT scaling, performance classification, practical consequences

## Abstract

In item response theory (IRT) models, assessing model-data fit is an essential step in IRT calibration. While no general agreement has ever been reached on the best methods or approaches to use for detecting misfit, perhaps the more important comment based upon the research findings is that rarely does the research evaluate IRT misfit by focusing on the practical consequences of misfit. The study investigated the practical consequences of IRT model misfit in examining the equating performance and the classification of examinees into performance categories in a simulation study that mimics a typical large-scale statewide assessment program with mixed-format test data. The simulation study was implemented by varying three factors, including choice of IRT model, amount of growth/change of examinees’ abilities between two adjacent administration years, and choice of IRT scaling methods. Findings indicated that the extent of significant consequences of model misfit varied over the choice of model and IRT scaling methods. In comparison with mean/sigma (MS) and Stocking and Lord characteristic curve (SL) methods, separate calibration with linking and fixed common item parameter (FCIP) procedure was more sensitive to model misfit and more robust against various amounts of ability shifts between two adjacent administrations regardless of model fit. SL was generally the least sensitive to model misfit in recovering equating conversion and MS was the least robust against ability shifts in recovering the equating conversion when a substantial degree of misfit was present. The key messages from the study are that practical ways are available to study model fit, and, model fit or misfit can have consequences that should be considered when choosing an IRT model. Not only does the study address the consequences of IRT model misfit, but also it is our hope to help researchers and practitioners find practical ways to study model fit and to investigate the validity of particular IRT models for achieving a specified purpose, to assure that the successful use of the IRT models are realized, and to improve the applications of IRT models with educational and psychological test data.

## Introduction

In item response theory (IRT) models, various methods and approaches have been suggested for detecting model misfit (Swaminathan et al., 2007), and these measures of model fit typically summarize the discrepancy between observed values and the values expected under an IRT model at either the item level or test level by statistical tests of significance and/or graphical displays.

While no general agreement has ever been reached on the best methods or approaches to use for detecting misfit, perhaps the more important comment based upon the research findings is that rarely does the research evaluate IRT misfit by focusing on the consequences of using misfitting items and item statistics and estimation errors associated with them. That is, does the amount of model misfit observed have a practical impact on the intended application? Ultimately, it is the consequences of the misfit that should be considered in deciding on the merits of a particular model for use in particular situations. If the consequences are minor, then the misfit in the model may be tolerable (Hambleton and Han, 2005).

### Consequences of IRT Misfit

Despite the importance of assessing the consequences of IRT misfit to practical decision-making, this research area has not been given much attention as it deserves in the IRT fit literature (Hambleton and Han, 2005). Recently, few empirical studies emerged. Sinharay and Haberman (2014) studied practical significance of model misfit with various empirical data sets and concluded that the misfit was not always practically significant though evidence of misfit for a substantial number of items was demonstrated. Zhao (2017) investigated the practical impact of item misfit with Patient-Reported Outcome Measurement Information System (PROMIS) depression and pain interference item banks, and suggested that item misfit had a negligible impact on score estimates and severity classifications with the studied sample. Meijer and Tendeiro (2015) analyzed two empirical data sets and examined the effect of removing misfitting items and misfitting item score patterns on the rank order of test takers according to their proficiency level score, and found that the impact of removing misfitting items and item score patterns varied depending on the IRT model applied. The above studies offer good examples of the assessment of the consequences of IRT misfit through examining the agreement of the decisions made based on including/excluding misfitting items (item misfit) or including/excluding misfitting person (person misfit).

In practice, however, it is sometimes less feasible to simply remove misfitting items. Even significance tests suggest an item lack of fit, the item may still need to be retained in the whole test, rather than being taken out, because, for instance, the item has to be kept for balancing content coverage or for IRT-based analyses, such as, IRT-based equating, differential item functioning, and ability estimates in a computer-adaptive testing environment. Further, when misfit items are deleted, test score reliability and validity are likely to be lowered. Similarly, it is impracticable to remove misfitting persons particularly for individual-level analyses in which ability parameter estimate is used for decision-making.

A third type of misfit, in addition to the item level and person level, occurs at the test level, which is also referred to as model misspecification. Assessment of misfit globally at the test level is not uncommon and is of great practical value in operational assessment programs (e.g., statewide or nationwide educational assessment). Like almost all statistical models, a perfectly fitting model rarely exists. “Fit is always a matter of degree (Tendeiro and Meijer, 2015).” Operationally, a model that does not show a decent fit might be practically usable in a particular application, and the misfitting (or, misspecified) model could still be preferred due to practical reasons, such as, model simplicity, software availability and cost/time effectiveness. With additional parameters added in to a model, a more complex model in a model hierarchy tends to fit better than a simpler model nested under it, but these parameters might appear to be less stable under replications (Molenaar, 1997). In other words, the decision of model selection involves a trade-off between model complexity/parameter stability and the degree of model-data fit. Furthermore, once a certain IRT model is determined in the beginning of the administration of an assessment program, such a model typically has to be sustained for item calibration, test equating and scoring in the subsequent administrations from year to year. The degree of misfit could be varied across administrations due to reasons like changes of latent trait distributions and item parameter drift (Park et al., 2016). Depending on the intended model uses, the practical consequences of misfit might or might not be tolerable. It is, therefore, always imperative to examine practical consequences of misfit and weight/balance the consequences against model advantages and intended applications. Among the limited body of literature that studied model misfit consequences at the test level, Lu (2006) conducted simulation studies to investigate the consequences of misfit on score differences in the applications of test equating with dichotomous data, and concluded that the two-parameter logistic model and the three-parameter logistic model performed equally well but the one-parameter logistic model resulted in considerable equating conversion errors. Few, if any, studies to date have been conducted on the focus of the test level misfit with mixed-format test data, which is a typical case in operational assessment programs nowadays.

### IRT Equating

Test equating secures the comparability of test scores across different test administrations/forms. IRT offers solutions for test equating, and IRT equating methods, compared with conventional methods, possess several practical advantages, such as, better solution for equating at the upper ends of score scales, greater flexibility in choosing test forms for equating, and possibility of item-level preequating (see Cook and Eignor, 1991, for details). IRT equating typically contains three steps: (a) selecting a data collection design, (b) placing parameter estimates on a common scale, and (c) equating test scores. The non-equivalent-groups anchor test (NEAT) design, also referred to as the common-item non-equivalent groups (CINEG) design, is commonly used due to its practical efficiency and feasibility. Among IRT equating methods (Kolen and Brennan, 2014), the mean/sigma (MS; Marco, 1977) and Stocking and Lord characteristic curve (SL; Stocking and Lord, 1983) methods are widely used. Alternatively, separate calibration with linking and fixed common item parameter (FCIP; Jodoin et al., 2003; Kim, 2006; Keller and Hambleton, 2013) has become popular in operational assessment programs. In IRT, once the common scale has been established, the equating of test scores across forms is typically performed using IRT true score equating, although alternative equating methods are available (e.g., observed score equating). More details about IRT scaling and equating methods can be found in Kolen and Brennan (2014).

Previous studies have shown that the SL method and the FCIP procedure performed similarly, and both outperformed the MS method in recovering ability changes (Pang et al., 2010; Keller and Keller, 2011; Keller and Hambleton, 2013). With dichotomous data, the characteristic curve methods performed better than the FCIP procedure when there was a mean shift in ability distribution (Keller and Keller, 2011); with mixed-format test data, however, FCIP performed best comparing to the characteristic curve methods (Keller and Hambleton, 2013).

As noted in previous equating studies (Keller and Keller, 2011; Keller and Hambleton, 2013; Kolen and Brennan, 2014), model fit is a strong assumption that IRT equating is based on. Only when the fit between the model and the empirical data of interest is satisfactory, can the IRT equating be appropriately applied. Despite the importance of model fit in the context of IRT equating, there appears to be limited research that examines the performance of IRT equating in the presence of model misfit/misspecification. Related studies addressed IRT equating performance on the violations of the IRT assumptions, for example, several studies (De Champlain, 1996; Bolt, 1999) demonstrated that IRT equating is fairly robust when the violation of the IRT unidimensioanlity assumption is not too severe. It is, however, unclear how various IRT scaling methods perform when misfit presents. The present study thus attempted to fill the gap in the literature through examining the performance of different IRT scaling methods in the presence of model misspecification. The findings would be of great practical value and interest, given that IRT equating is a routine practice and the misfit situations are inevitably encountered in the operational assessment programs.

### Overview of the Present Study

To put the consequence issue into perspective, we illustrate an example here using two adjacent administrations of a statewide assessment program. Choice of model, either a well-fitting or a poor-fitting IRT model, resulted in up to a two score point difference and impacted up to about 1000 (2%) examinees being placed into different pass/fail classifications. In short, the use of a poor-fitting IRT model made substantial differences of the score conversions and resulted in moderate differences of the performance classifications in this case.

Further to the above real data analysis, we considered Monte Carlo simulations in the present study, where true item and ability parameters were known and they were used to generate examinee response data, so as to provide a basis that the results derived by different models (either well-fitting or misfitting) could be compared to. Moreover, with a simulation design, different degrees of model misfit and potential factors that might affect the assessment of misfit consequences could be conventionally manipulated, such that the validity of inferences drawn from various degrees of model misfit can be investigated.

To sum up, the purpose of the study was to investigate the consequences of IRT model misfit under three IRT scaling methods through a simulation study that mimics two adjacent administration years in a typical statewide assessment program. We considered two consequence measures: (a) equating performance and (b) classification of examinees into performance categories, given the following reasons. Equating is a common practice and an essential issue in operational assessment programs. Additionally, the percentage of examinees being classified into different performance categories, in particular, the percentage of examinees at or above the proficiency level, is a commonly used reporting measure for accountability, and serves as important indicators in the statewide assessment programs.

## Materials and Methods

### Data Sources

A simulation study was conducted based on realistic item parameters obtained from a mathematics examination of a high-stakes statewide assessment program. The program adopts a matrix-sample external anchor equating design and employs mixed-format test data which contain dichotomously scored multiple choice (MC) items (0/1 scored), dichotomously scored short answer (SR) items (0/1 scored), and polytomously scored constructed response (CR) items (0–4 scored). Two adjacent administration years, namely reference year and new year, were focused. Each administration contained 39 unique operational items (which were used for scoring examinees) and 39 external anchor items (which were common across the two administrations, and did not contribute to scoring the examinees).

### Simulation Design and Factors

The simulation study was implemented by varying three factors: (a) choice of IRT model, (b) amount of growth/change of examinees’ abilities between two adjacent administration years, and (c) choice of IRT scaling methods/procedures.

With respect to the choice of IRT model, we considered one-, two-, and three-parameter logistic models (1PL, 2PL, and 3PL, respectively) for dichotomous data, and generalized partial credit model (GPC; Muraki, 1992) and partial credit model (PC; Masters, 1982) for polytomous data. On a related note, the graded response model (GRM; Samejima, 1969) was not considered in the study given that little evidence in the measurement literature showed obvious difference of results by using GRM and GPC (Nering and Ostini, 2010).

Regarding the second factor, growth/change of examinees’ abilities was built by varying the changes in the means of examinees’ ability distributions between the reference and new years. Four conditions of mean shift were built into the simulation: 0.00, 0.10, 0.25, and 0.50, on the ability (𝜃, theta) scale. The mean shift of 0.00 represented no change. The mean shift of 0.10 reflected common growth/change of examinees’ abilities between two adjacent years in a statewide assessment programs (e.g., Jodoin et al., 2003; Keller and Hambleton, 2013), and the amount of change was regarded as moderate. The mean shifts of 0.25 and 0.50 were sizable, and they were considered relatively large.

In terms of the third factor, three IRT scaling procedures were implemented in the simulation study for placing item parameter estimates on a common scale: mean/sigma (MS), Stocking and Lord characteristic curve method (SL), and separate calibration with linking and FCIP. More details of each procedure will be described shortly.

### Data Generation

Realistic item parameter estimates obtained from the item calibration on the operational assessment program were taken for data simulation. The 3PL (for MC items), 2PL (for SR items) and GPC (for CR items) model set (3PL/2PL/GPC) was determined for the use of the real data calibration and thus was utilized for generating item responses of the mixed-format data in the simulation study. Slight dimensionality might be potentially affected by different item types (Thissen et al., 1994); however, findings from previous studies (e.g., Drasgow and Parsons, 1983; Reckase et al., 1988) demonstrated that unidimensional models are fairly robust to small amounts of multidimensionality. We thus decided to use the unidimensional model set 3PL/2PL/GPC as the generating model.

Each administration contained 39 unique items (including 29 0/1 scored MCs, 5 0/1 scored SRs and 5 04 scored CRs; total raw score = 54), and 39 anchor items (consisting of 29 0/1 scored MCs, 5 0/1 scored SRs, and 5 04 scored CRs) that were common across the two administrations, so as to mimic the realistic compositions of mixed-format test data in the operational assessment program. A NEAT design was adopted (a common choice in state equating work), where a cohort of examinees in the reference year took all 39 items of Form X, and another cohort of examinees in the new year took all 39 items of Form Y. All examinees of both administrations took the 39-item anchor set, Form A.

In the reference year administration (Form X+A), a standard normal distribution of true ability (i.e., 𝜃_XA_ *∼N* (0.00, 1.00)) was generated. In the new year administration (Form Y+A), four conditions of the true ability distributions were implemented: 𝜃_Y A_ *∼N* (0.00, 1.00), 𝜃_Y A_ *∼N* (0.10, 1.00), 𝜃_Y A_ *∼N* (0.25, 1.00) and 𝜃_Y A_ *∼N* (0.50, 1.00), for introducing various amounts of ability changes or growth between the two adjacent administrations by varying mean shifts but keeping the standard deviation as a constant.

The computer program WINGEN3 (Han, 2007) was used to simulate the item responses. A large sample of examinees (*N* = 50,000) was generated for each dataset in each administration to reflect the realistic number of examinees in the operational assessment program. 50 datasets were generated for each of the five test forms (i.e., 𝜃_XA_ *∼N* (0.00, 1.00) on Form X+A; 𝜃_Y A_ *∼N* (0.00, 1.00), 𝜃_Y A_ *∼N* (0.10, 1.00), 𝜃_Y A_ *∼N* (0.25, 1.00) and 𝜃_Y A_ *∼N* (0.50, 1.00) on Form Y+A), applying the same set of item and ability parameters for simulation.

### Data Calibration

For each simulated dataset, three combinations of dichotomous and polytomous models were applied for calibrating the item responses: (a) the model set of 3PL, 2PL, and GPC (3PL/2PL/GPC), (b) the model set of 2PL, 2PL, and GPC (2PL/2PL/GPC), and (c) the model set of 1PL, 1PL, and PC (1PL/1PL/PC). The more constrained model set is nested within the less restricted model set. Such a design of calibration models has been used in other model fit simulation studies such as Chon et al. (2010).

Item parameters for the mixed-format datasets were calibrated using the computer program PARSCALE (Muraki and Bock, 2003). Along with the default options of PARSCALE, the following optional commands were executed: TPRIOR, GPRIOR, and SPRIOR. 60 quadrature points of ability, equally spaced from –4 to 4, were used. To increase the precision of calibration and the possibility of convergence, the convergence criterion was set to 0.001 and the maximum number of expectation-maximization (EM) cycles attempted to reach convergence was increased to 200. Not surprisingly, convergence failed in some replications when 2PL/2PL/GPC and 1PL/1PL/PC were used to calibrate the response data, because of the presence of one or more seriously misfitting item(s) due to the choice of misspecified IRT models. Such a replication was discarded and a new replication was re-generated until a total of 50 replications were reached and all items in each replication were successfully calibrated by all three sets of calibration models (i.e., 3PL/2PL/GPC, 2PL/2PL/GPC, and 1PL/1PL/PC).

### IRT Scaling and Test Equating

After the separate calibration, three IRT scaling methods, namely, MS, SL, and FCIP, were applied independently to place the item parameter estimates of the new year administration onto the scale of the reference year administration. All three IRT scaling methods were performed under each calibration model set within each ability shift condition.

Mean/sigma and SL are linear transformation methods that utilize separately estimated anchor item parameters taken from the reference and new year administrations to determine the scaling constants for placing item parameter estimates on a common scale. The MS method uses the means and the standard deviations of the anchor item *b*-parameter estimates (or ability estimates) from each administration to obtain the scaling constants; whereas the SL method identify the scaling constants such that the differences between the test characteristic curves (TCCs) of the anchor set between the two administrations are minimized. A sum of squared differences between the TCCs of the two administrations in the criterion function were divided at 60 equally spaced quadrature points between –4 and 4 at the theta scale for minimizing the differences. The computer program STUIRT (Kim and Kolen, 2004) was used for applying the MS and SL procedures.

In the FCIP procedure, the new year dataset (Form Y+A) was calibrated in PARSCALE with the item parameters for anchor items (on Form A) fixed to values of those from the reference year administration, resulting in placing all item parameters on a common metric. The command options of POSTERIOR and FREE = (NOADJUST, NOADJUST) were added in the PARSCALE syntax, as suggested by Kim (2006), for more accurate recovery of the ability parameters.

After parameter estimates for the two test forms (Forms X and Y) were placed on a common scale, true score equating was performed for equating the expected scores (also referred to as TCC score, or expected true score) on the two test forms so that comparisons across calibration models can be made. The computer program POLYEQUATE (Kolen, 2004) was used for performing the IRT true score equating and establishing score conversions based on the expected scores between Form X and Form Y. The expected scores on the two forms in the conversions were regarded as equated scores.

### Evaluation Criteria

Consequences of misfit were evaluated in terms of (a) the recovery of true score conversion resulting from equating and (b) the recovery of classifications of examinees into performance categories in comparison with the truth.

#### Equating Performance

The true conversion table based on the true 3PL/2PL/GPC item parameters of Forms X and Y (on the same scale) was expected to reveal the true relationship of the equated scores between the two test forms. With the simulated examinee response data, a total of 1800 estimated conversion tables (3 calibration models × 3 IRT scaling methods × 4 ability shifts × 50 replications) were produced covering all 36 simulated conditions. In order to examine the capability of the true score conversion recovery, we computed the mean errors of equating (MEEs) and the root mean square errors of equating (RMSEEs) between the estimated and the true conversion tables conditioning at each number of correct (NC) score point under each condition. According to Kolen and Brennan (2014), there are generally two sources of equating errors: random equating error (such as due to sampling) and systematic equating error (such as due to estimation methods). Given the notion that sampling error can be ignored when the samples are large enough (Dorans et al., 2010), we considered the systematic equating error resulting from model misfit as the major source of equating error of interest in the study.

In addition to the individual score point level, we calculated root expected mean square error of equating (REMSEE) as a single aggregate measure/indicator for the recovery of the true equating conversion under each condition. The REMSEE is an equally weighted average of the RMSEEs over NC score points. We used the difference that matters (DTM; Dorans and Feigenbaum, 1994), which is defined as any REMSEE that is equal to or greater than 0.5 (half a NC score point), to judge whether the REMSEE is of practical significance. DTM is often used as practical guideline and was adopted in other equating studies, such as, Kim and Walker (2011).

#### Classification of Examinees into Performance Categories

In the operational assessment program that the study is based on, examinees’ performances are classified in four categories: “inadequate”, “adequate”, “proficient”, and “advanced”^1^, applying the cut-off points of 21, 37, and 49, respectively on the NC score metric on Form X, according to standard setting conducted operationally. The four performance categories were converted into pass and fail categories by applying the cut score of 37. The corresponding cut-off scores on Form Y were determined by identifying the three equated scores on Form Y that were, respectively, linked to the NC scores of 21, 37, and 49 on Form X in the corresponding conversion tables.

Under each condition, two performance classifications were computed for each examinee: true classification (PC_true_) and estimated classification (PC_estimated_), where the former represented the performance category that an examinee was classified into using his/her true equated score, and the latter was estimated based on equated score. An examinee was considered as being accurately classified if PC_estimated_ = PC_true_, as being over-classified if PC_estimated_ > PC_true_, and as being under-classified if PC_estimated_ < PC_true_. Under each condition, classification accuracy, over-classification rate and under-classification rate were calculated by averaging the percentages of respective examinees over 50 replications. Furthermore, passing rate was computed by calculating the percentage of examinees being classified as “proficient” and “advanced” in each replication. Passing misclassification, the difference between the estimated and the true passing rates, was computed for each condition. A positive value of passing misclassification suggested that the true passing rate was over-estimated, and a negative value implied that the true passing rate was under-estimated.

## Results

The consequences of misfit were evaluated on equating performance and classification of performance categories, with results reported below. When 3PL/2PL/GPC was used as the calibration model (calibration model = generating model), negligible degree of misfit was expected. When 2PL/2PL/GPC and 1PL/1PL/PC were employed as the calibration model (calibration model ≠ generating model), minor and sizable degrees of misfit were expected to be present, respectively.

### Equating Performance

**FIGURE 1:**
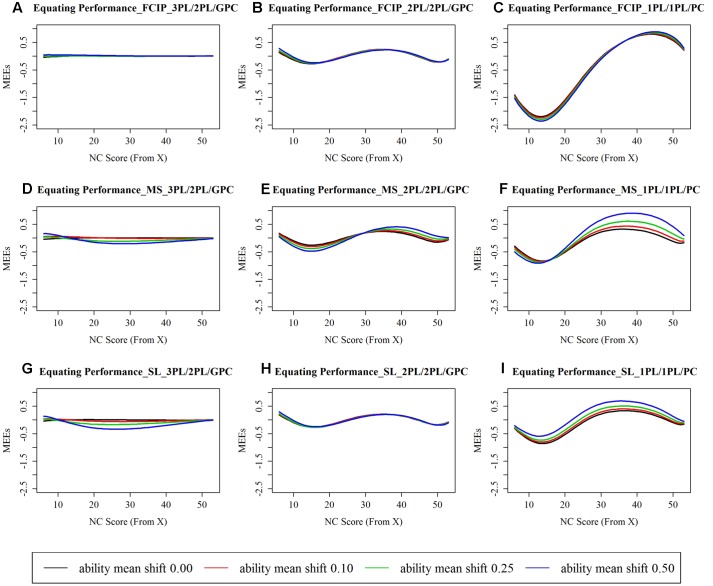
**Mean errors of equating (MEE) between estimated and true score conversions. (A)** Equating performance under the 3PL/2PL/GPC × FCIP condition; **(B)** Equating performance under the 2PL/2PL/GPC × FCIP condition; **(C)** Equating performance under the 1PL/1PL/PC × FCIP condition; **(D)** Equating performance under the 3PL/2PL/GPC × MS condition; **(E)** Equating performance under the 2PL/2PL/GPC × MS condition; **(F)** Equating performance under the 1PL/1PL/PC × MS condition; **(G)** Equating performance under the 3PL/2PL/GPC × SL condition; **(H)** Equating performance under the 2PL/2PL/GPC × SL condition; **(I)** Equating performance under the 1PL/1PL/PC × SL condition. FCIP, separate calibration with linking and fixed common item parameter; MS, mean/sigma method; SL, Stocking and Lord characteristic curve method; 3PL, three-parameter logistic model; 2PL, two-parameter logistic model; 1PL, one-parameter logistic model; GPC, generalized partial credit model; PC, partial credit model. *x*-axis, number of correct (NC) score point ranging from 6 to 53. The examinees being scored out of this range were those who answered all items correct or answered quite fewer items correct than simply guessing based on the generating model. *y*-axis, mean errors of equating (MEEs) between the estimated and the true conversion tables conditioning at each NC score point.

**Figure 1** displays the MEEs along NC scores from 6 to 53, and **Table 1** reports the REMSEE under each studied condition. The examinees being scored out of the range between 6 and 53 were those who answered all items correct or answered quite fewer items correct than simply guessing based on the true 3PL/2PL/GPC model. As can be seen in **Figure 1**, 3PL/2PL/GPC produced almost identical score conversions compared to the true conversion, 2PL/2PL/GPC resulted in a small amount of discrepancies on average between the estimated score conversions and the truth, and 1PL/1PL/PC produced the largest conversion errors compared to the other two calibration models, regardless of choice of IRT scaling methods and various amounts of ability shifts.

**Table 1:** Root expected mean square error of equating, performance misclassifications, passing rates, and passing misclassifications between estimation and truth.

Ability shift	True passing Rate (%)	Models	IRT scaling methods^a^	REMSEE^b^	Classification Misclassifications (%)^c^		Passing rates (%)	Passing misclassifications (%)^d^
					UC	OC		
0.00	50.72	3PL/2PL/GPC	FCIP	0.04	5.99	6.20	50.82	0.09
			MS	0.10	5.97	6.23	50.83	0.11
			SL	0.04	5.94	6.25	50.85	0.12
		2PL/2PL/GPC	FCIP	0.16	5.88	6.46	50.55	-0.18
			MS	0.16	5.96	6.38	50.49	-0.23
			SL	0.15	5.85	6.49	50.58	-0.14
		1PL/1PL/PC	FCIP	1.02	7.19	6.49	49.74	-0.98
			MS	0.35	6.45	6.48	50.04	-0.68
			SL	0.36	6.42	6.54	50.04	-0.68
0.10	54.61	3PL/2PL/GPC	FCIP	0.05	6.12	6.19	54.71	0.09
			MS	0.09	6.06	6.26	54.74	0.12
			SL	0.06	5.96	6.34	54.82	0.21
		2PL/2PL/GPC	FCIP	0.16	6.00	6.44	54.41	-0.20
			MS	0.18	6.04	6.40	54.34	-0.27
			SL	0.15	5.80	6.65	54.54	-0.07
		1PL/1PL/PC	FCIP	1.04	7.57	6.23	53.62	-1.00
			MS	0.39	6.90	6.33	53.95	-0.67
			SL	0.37	6.65	6.41	53.95	-0.67
0.25	60.59	3PL/2PL/GPC	FCIP	0.05	6.23	6.11	60.60	0.01
			MS	0.12	5.93	6.42	60.78	0.19
			SL	0.12	5.86	6.48	60.85	0.26
		2PL/2PL/GPC	FCIP	0.16	6.12	6.35	60.29	-0.30
			MS	0.21	6.16	6.33	60.17	-0.42
			SL	0.15	5.66	6.83	60.53	-0.06
		1PL/1PL/PC	FCIP	1.07	8.23	5.76	59.48	-1.11
			MS	0.48	8.08	5.69	59.82	-0.77
			SL	0.38	7.02	6.13	59.82	-0.77
0.50	69.73	3PL/2PL/GPC	FCIP	0.05	6.38	5.79	69.60	-0.13
			MS	0.17	5.83	6.34	69.89	0.15
			SL	0.20	5.65	6.49	70.08	0.35
		2PL/2PL/GPC	FCIP	0.16	6.24	6.03	69.30	-0.43
			MS	0.27	6.24	6.04	68.76	-0.98
			SL	0.15	5.37	6.94	69.17	-0.56
		1PL/1PL/PC	FCIP	1.11	9.07	4.91	68.54	-1.19
			MS	0.63	9.43	4.59	67.98	-1.75
			SL	0.43	7.44	5.53	68.89	-0.84

*^a^FCIP = separate calibration with linking and fixed common item parameter; MS = mean/sigma method; SL = Stocking and Lord characteristic curve method.*

*^b^REMSEE = Root Expected Mean Square Error of Equating.*

*^c^UC = under-classificationOC = over-classification.*

*^d^Passing misclassification = the difference between the estimated and the true passing rates, where a positive sign (+) represents an over-estimated passing rate and a negative sign (–) represents an underestimated passing rate.*

In addition to the magnitude, the estimation direction is also noteworthy. As can be seen in **Figure 1**, 2PL/2PL/GPC underestimated conversion errors for lower NC scores, overestimated conversion errors for middle to high NC scores, and underestimated conversion errors for higher end of NC scores; while, 1PL/1PL/PC underestimated conversion errors for low to middle NC scores, and overestimated conversion errors for middle to high NC scores. The findings discussed above are in line with the TCC performance. Taking the condition of FCIP × ability shift of 0.50 as an illustrated example, due to the absence of the *c* parameters in the 2PL and 1PL, the 2PL/2PL/GPC TCC underestimated the true TCC along the lower end of 𝜃s (below –1), slightly overestimated the truth along –1 < 𝜃 < 0.5, and slightly underestimated the truth for 𝜃s greater than 0.5 roughly. The 1PL/1PL/PC TCC underestimated the true TCC along the lower end of *𝜃*s (below –1), and slightly overestimated it along 𝜃s approximately greater than –1.

In terms of the REMSEE, as can be seen in **Table 1**, under 3PL/2PL/GPC, FCIP, and SL performed equally well and MS performed least well when there was a moderate amount of ability shift (mean shift of 0.10); and, SL and MS performed similarly less well than FCIP when there was a sizable amount of ability shift (mean shifts of 0.25 and 0.50). Under 2PL/2PL/GPC, FCIP, and SL performed similarly under all ability shift conditions and MS produced largest conversion errors than FCIP and SL when there was a sizable amount of ability shift (mean shifts of 0.25 and 0.50).

Under 1PL/1PL/PC (see **Table 1**), MS and SL produced smaller conversion errors than FCIP regardless of amount of ability shifts. The REMEEs under all 1PL/1PL/PC × FCIP conditions were above one score point, two times of the DTM, regardless of various amounts of ability shifts, implying that the impact of the choice of 1PL/1PL/PC on equating using the FCIP procedure was practically significant. In addition, MS generally produced larger conversion errors than SL when the ability shifts were increased from 0.10 to 0.25 and 0.50. In particular, the REMEE was larger than 0.50 score points, the DTM, under the condition of 1PL/1PL/PC × MS × ability mean shift of 0.50.

Taken together, 2PL/2PL/GPC performed slightly less well than 3PL/2PL/GPC, as expected, but its impact on equating was not practically significant, given that the resulting average conversion errors were below the DTM. 1PL/1PL/PC performed much less well than 3PL/2PL/GPC and 2PL/2PL/GPC, regardless of the choice of IRT scaling methods and various amounts of ability shifts. In particular, under 1PL/1PL/PC, when FCIP was implemented, the conversions errors were consistently large and the consequence of misfit was practically significant in all ability shift conditions.

### Classification of Examinees into Performance Categories

The misfit consequences were further assessed in classification accuracy, performance category misclassification, and passing misclassification. Classification accuracies ranged from 85.98 to 87.85% under all conditions, implying that all calibration models, either well-fitting or misfitting ones, produced a high degree of agreement of performance classifications compared to the truth. The absolute values of passing misclassifications ranged from 0.01 to 1.75% (see **Table 1**).

With the choice of 3PL/2PL/GPC, all three IRT scaling methods produced closely identical classification accuracies, and generally overestimated the passing rate (shown as positive values of passing misclassifications), with the exception of FCIP in the condition of 0.50 ability mean shift. FCIP resulted in the smallest passing misclassification amongst the three scaling methods no matter how much mean ability shifted. SL produced slightly greater passing misclassification than MS when the amount of ability shifts was moderate or sizable (mean shifts of 0.10, 0.25, and 0.50).

With the choice of 2PL/2PL/GPC, all three IRT scaling method produced roughly similar classification accuracies, and consistently underestimated passing rates regardless of ability shifts (shown as negative values of passing misclassifications). In recovering the passing rates, MS performed least well in all ability shift conditions, and SL performed better than FCIP with the exception of the condition of 0.50 ability mean shift.

With 1PL/1PL/PC being the calibration model, SL generally produced the highest classification accuracies and FCIP largely produced the lowest classification accuracies when there was a moderate or sizable amount of shift in ability. Additionally, under-classification rates were higher than over-classifications rates regardless of choice of IRT scaling methods when there were moderate or sizable amounts of ability shift. Regarding passing rates, all three IRT scaling method underestimated passing classification regardless of ability shifts. FCIP produced the greatest departure from the true passing rate irrespective of ability shifts. In general, SL outperformed MS in recovering passing classification when the amount of ability shift was moderate or large. In particular, MS was the least capable of recovering passing classification when the ability shift was substantial (a mean shift of 0.50).

In sum, when FCIP was performed, 1PL/1PL/PC produced lower classification accuracies than the others regardless of amount of ability shifts. Similar to the equating findings, 1PL/1PL/PC × FCIP produced greater passing misclassifications under all conditions of ability shifts. When the MS was implemented, out of 50,000 examinees, the choice of 1PL/1PL/PC model impacted up to 875 examinees (1.75%, the largest passing misclassification) being misclassified as fail when the mean ability shift was 0.50.

## Discussion

Findings indicated that the extent of significant consequences of model misfit varied over the choice of model and IRT scaling method. A detailed discussion follows.

### Consequences of Model Selection

Under 3PL/2PL/GPC (calibration model = generating model), the estimation results were very close to the truth as expected, because no model misfit was introduced and the discrepancies of the estimation from the truth were mainly due to random sampling error, random equating error and scale shift. It should be noted that the scale for the estimated parameters was not exactly the one for the true parameters. The reason why we avoided placing the estimated parameters on the same scale as the true parameters is that we considered scale shift as part of the consequences of model fit/misfit. How much the scale shifts are usually unknown operationally, because the true scale is unknown. The scale shift was reserved under all simulation conditions in the present study such that the consequence measures due to model selection, regardless of the use of well-fitting or misfitting models, can be comparable.

When 2PL/2PL/GPC was employed as the calibration model, where a minor degree of misfit was introduced, its consequences on the equating performance, the passing rate, and the mapping of performance categories were not practically significant. It should be noted, however, that only the 29 multiple-choice items were calibrated by the 2PL which is different from the generating model, and the remaining 10 items (five short answer items and five constructed response items) were calibrated by the same models as the generating model.

When 1PL/1PL/PC was utilized as the calibrating model, the greatest discrepancies of the estimations from the truth were identified, in particular, when FCIP was implemented. Notably, the consequences of using 1PL/1PL/PC on the FCIP equating performance were significant regardless of amount of ability shift between reference and new years. Moreover, substantial misclassifications of passing rates and performance categories were produced under all conditions of ability shifts. When MS was employed under the condition of sizable ability shift, the consequence of using the 1PL/1PL/PC model on equating performance was significant and it produced the largest passing misclassification amongst all conditions.

### IRT Scaling Methods in the Presence of Model Fit and Misfit

The present study also yielded useful findings on the capability of the three IRT scaling methods in the presence of model fit and misfit under various ability shift conditions.

With the choice of the well-fitting model (3PL/2PL/GPC in the study), FCIP (REMSEE ranged from 0.04 to 0.05) appeared to be slightly more robust than MS (REMSEE ranged from 0.09 to 0.17) and SL (REMSEE ranged from 0.04 to 0.20) in capturing various ability shifts and performed best when there was a small or sizable amount of ability shift. Regarding the equating conversion recovery, MS performed worse than FCIP and SL when there was zero or small amount of ability shift. SL and MS performed similarly less well than FCIP when there was a substantial amount of ability shift. Regarding the passing rates recovery, FCIP outperformed the others regardless of ability shifts. SL produced slightly greater passing misclassifications than MS when there was a moderate or sizable amount of ability shift. To a great extent, our findings are not in opposition to the results of related studies (Pang et al., 2010; Keller and Keller, 2011; Keller and Hambleton, 2013).

In the presence of model misfit (2PL/2PL/GPC and 1PL/1PL/PC in the study), when a minor degree of misfit (2PL/2PL/GPC in the study) was present, FCIP (REMSEE = 0.16) and SL (REMSEE = 0.15) were more robust against various amounts of ability shifts than MS (REMSEE ranged from 0.16 to 0.27) in recovering the equating conversion, and FCIP and SL outperformed MS in recovering passing rates in all ability shift conditions. When a substantial degree of misfit (1PL/1PL/PC in the study) was present, FCIP (REMSEE ranged from 1.02 to 1.11) and SL (REMSEE ranged from 0.36 to 0.43) were found to be generally robust in recovering equating conversions, and MS was the least robust against different amount of ability shifts in recovering the equating conversion (REMSEE ranged from 0.35 to 0.63). FCIP produced the greatest conversion errors and passing misclassifications regardless of ability shifts. SL performed best in recovering the equating conversion when there was a moderate or substantial amount of ability shifts, and MS performed least well in recovering passing rates when the amount of ability shift was substantial.

It is worth highlighting that, among the three IRT scaling methods, FCIP performed best in recovering passing rates and in capturing various amounts of ability shifts when the model fitted the data. Its capability of capturing ability shifts under the well-fitting model lends support for the PARSCALE syntax refinement suggested by Kim (2006). Kim presented several alternatives for the FCIP calibration and suggested using POSTERIOR option, which allows the prior distributions to be updated after the EM cycles, and suggested specifying the FREE option, which prevents the rescaling of the parameters during the EM cycles. On the other hand, with a large degree of model misfit, FCIP performed least well in recovering the equating conversion and the passing classification. As Kim (2006) and Yen and Fitzpatrick (2006) discussed, FCIP forces PARSCALE to accommodate the anchor item parameter values by fixing them to the values of the anchor item parameters obtained from the reference year administration. Therefore, FCIP can adversely affect the item calibration when the model does not fit the data closely. In other words, FCIP is sensitive to model misfit, and model fit is critical to the appropriate use of FCIP. When the calibration model is wrong, FCIP can produce significant consequences.

To sum up, in comparison with MS and SL, FCIP was more sensitive to model misfit and more robust against various amounts of ability shifts between two adjacent administrations regardless of model fit. SL was generally the least sensitive to model misfit in recovering equating conversion and MS was the least robust against ability shifts in recovering the equating conversion when a substantial degree of misfit was present.

### Recommendations of Good Practices for Investigating Model Misfit Consequences

We believe that it is necessary to reiterate the need to address the consequences of model misfit for intended applications that testing agencies and practitioners in mind. Practitioners or researchers who study IRT model fit often carry out model fit analysis solely based on statistical significance testing and ignore the step of directly addressing the practical consequences of model misfit. As uniformly important as assessing statistical significance of model fit, assessing the practical significance of model misfit is a necessary step of model fit evaluation, because some model misfit with some particular applications may be quite bearable. Below we seek to provide our views for good practices for investigating model misfit consequences. We hope that practitioners and researchers interested in studying consequences of IRT model misfit will find this helpful.

First, we advocate that investigating consequences of model misfit for intended applications should be routine for testing agencies and practitioners. Model fit or misfit can have consequences that should be considered in choosing a model. For example, if sample size is very large, even a small amount of discrepancies between the data and model can suggest statistically significant model misfit, but the nature of the misfit may be of insignificant practical consequence. In another example, large practically meaningful levels of misfit might not be detected by goodness-of-fit test statistics with small samples. Even acceptable fit is suggested by statistical significance tests, there is still the need for the investigation of the practical consequences of model utilization.

Additionally, we would recommend that the examination of model misfit consequences should involve multiple procedures. As discussed earlier, it is sometimes less practicable to simply remove misfitting items which play central role, such as, in content balance and inclusion for IRT-based analyses. The assessment of model misfit consequences should go well beyond item-level analysis and involve a test-level evaluation when appropriate. Practitioners and researchers are recommended to looking at the practical consequences of choosing one model over another in their work, weight the consequences against model advantages and intended applications, and judge the practical significance of any differences before making decisions about actions to take with the misfitting items/models.

Last but not least, we would suggest that the investigation of consequences of model misfit should directly address with each specific intended application using practically meaningful criterion. In the present study, we focused on two types of applications: recovery of equating performance and classification of examinees into performance categories. In each application, practical criteria were given to judge whether any differences observed were practically meaningful, and they offered a much better indication to judge practical implications against, such as, differences in the standard error of equating or differences in the parameter estimates. The message is that practical ways are available to study model misfit consequences with specific intended applications, and it is critical to clearly define a practically meaningful criterion for each application. Furthermore, the assessment of model misfit consequences should not be limited to one specific application; rather, it is an ongoing process to investigate the consequences of misfit with various applications and to accumulate all possible evidence to provide a sound psychometric basis for supporting the appropriateness of particular IRT models to its intended uses.

### Limitations and Future Directions

Limitations exist in the current study. The results shown here were slightly biased in favor of models used to generate the simulation data. To reduce bias, future studies could be conducted with datasets generated by either 2PL/2PL/GPC or 1PL/1PL/PC models and then calibrated and equated using the same procedures applied in the study. Moreover, the study could be extended, for instance, by manipulating the shifts of variance and the changes of the skewness in the ability distributions between administrations. Particularly, the MS procedure uses the mean and standard deviation of the anchor item parameters to determine the scaling constant, and thus the changes of the skewness in the examinee ability distributions play a key role in affecting the slope of the linear transformation function (Keller and Keller, 2011). Another factor worth considering in future studies is the effect of sample size. A large sample size was used in this study to mimic a typical operational statewide assessment program, and the stability and convergence of estimates were of less concern. For some other assessment programs targeting to smaller populations, it would likely be producing larger estimation errors, in particular when the misfitting model is applied. Last but not least, a single set of realistic item parameters was considered in the present study, which limits the generalizability of the results.

### Final Remarks

The key messages from the study are that practical ways are available to study model fit, and model fit or misfit can have consequences that should be considered in choosing a model. Not only does the study address the consequences of IRT model misfit, but also it is our hope to help researchers and practitioners find practical ways to study model fit and to investigate the validity of particular IRT models for achieving a specified purpose, to assure that the successful use of the IRT models are realized, and to improve the applications of IRT models with educational and psychological test data.

## Author Contributions

YZ initiated the study design, simulated the data, performed the data analysis, and wrote up the manuscript. RH substantially contributed to the conception of the study and critically revised the manuscript for important intellectual content.

## Conflict of Interest Statement

The authors declare that the research was conducted in the absence of any commercial or financial relationships that could be construed as a potential conflict of interest.

## Footnotes

1 The name of each performance level is not exactly the one used in the operational program, due to a confidentiality restriction.
